# Recognition of the primary stressors affecting intensive care patients: a systematic review

**DOI:** 10.1186/cc12480

**Published:** 2013-03-19

**Authors:** S Birch, S Elliot

**Affiliations:** 1 Leeds Teaching Hospitals, Leeds University Teaching Hospitals Trust, Leeds, UK

## Introduction

Intensive care patients suffer psychological and physiological distress that may have debilitating and long-lasting effects [1-3]. Healthcare professionals are in a position to help avoid or alleviate this stress [4]. To action this it is important to identify the main stressors from the patient's perspective. A systematic review was performed to provide a list of what patients consider stressors in intensive care. These were then ranked in order to provide an identification tool that can be used to shape appropriate care.

## Methods

A systematic review was performed using MEDLINE, CINAHL, Psych INFO and Academic Search Complete. Grey literature was included and searches were not restricted to type of intensive care or country. Criteria were used to filter those articles that identified the patients' views of their stressor, not the patient experience. Eligible articles were critiqued using the Critical Appraisal Skills Programme for qualitative studies [5] and brought together using a narrative synthesis. All of the reviewed studies used a questionnaire as a means to identify what elements on the intensive care patients found stressful. A list of the top-10 stressors could then be expressed for each study and compared. From this information, a set of guidelines for best practice were devised.

## Results

A total 1,424 articles were systematically assessed for suitability and applicability. Of these, 14 articles remained eligible for review. The stressors were ranked by their frequency in the individual studies' top-10 lists. In rank order: 1, Tubes. 2, Pain. 3, Sleep Difficulties. 4, Thirst. 5, Lack of Patient Understanding. Overall, stressors were found to be similar throughout all of the studies.

## Conclusion

The review identified a list of the most pertinent common stressors. Awareness of these and ranking in priority may enable plans of care to be instigated to effectively alleviate patient stress.
